# Risk Assessment and Prevention of Foot-and-Mouth Disease Transmission from Laos to China

**DOI:** 10.3390/vetsci12020092

**Published:** 2025-01-24

**Authors:** Jige Xin, Sixian Lan, Jun Ai, Bangquan Zeng, Aiguo Xin, Lingling Ye, Weidong Zuo, Yanlin Li, Diangang Han

**Affiliations:** 1 College of Veterinary Medicine, Yunnan Agricultural University, Kunming 650201, China; 2007032@ynau.edu.cn (J.X.); lsx08180818@163.com (S.L.); y1186744981@163.com (Y.L.); 2 Technology Center of Kunming Customs, Kunming 650200, China; 13769143048@163.com (J.A.); ye_ling08@sina.com (L.Y.); 3 Yunnan Provincial Center for Animal Disease Control and Prevention, Kunming 650201, China; quanbangzeng123@163.com; 4 National Foot-and-Mouth Disease Para-Reference Laboratory (Kunming), Yunnan Academy of Animal Husbandry and Veterinary Sciences, Kunming 650224, China; aiguo_xin@hotmail.com; 5 Comprehensive Technology Center of Mengla Customs, Mengla 666399, China; zuozwd@126.com

**Keywords:** foot-and-mouth disease, risk assessment, Laos, border, control measures

## Abstract

Foot-and-mouth disease (FMD) is classified as a Class I animal disease in China and listed as one of the notifiable animal diseases by the World Organization for Animal Health (WOAH). It significantly impacts the safe production of livestock and the trade of animals and related products. China’s Yunnan Province shares a 710 km border with Laos, with frequent cross-border trade, and the cross-border flow of animals and related products occurs from time to time. In order to prevent the introduction of FMD from the border areas of Laos into China, this study conducted an assessment of the entry, exposure, and consequences of FMD transmission. The findings revealed a “high” risk in terms of entry assessment, a “medium” risk in exposure assessment, and a “high” risk in the consequence assessment. Based on these assessments, the overall risk level for the introduction of FMD from Laos into China is determined to be “high”. Therefore, it is recommended that management measures are implemented, such as restricting animal movement across borders and strengthening inspection procedures for animals entering China, to effectively prevent FMD introduction from Laos.

## Simple Summary

Foot-and-mouth disease is an acute, febrile, and highly contagious disease caused by the foot-and-mouth disease virus, mainly affecting cloven-hoofed animals, such as cattle, buffalo, yak, pigs, camels, sheep, and goats. Foot-and-mouth disease is endemic in various regions of Asia, Africa, the Middle East, and Latin America. It significantly impacts the safe production of livestock, the trade of animals, and related products. The primary modes of transmission for foot-and-mouth disease virus are through the respiratory and digestive tracts. The movement of infected animals, animals in the incubation period, and carriers of the latent virus play a significant role in both the domestic and cross-border spread of foot-and-mouth disease virus. China’s Yunnan Province shares a 710 km border with Laos, with frequent cross-border trade, and the cross-border flow of animals and related products occurs from time to time. This study conducted an assessment of the entry, exposure, and consequences of foot-and-mouth disease transmission. Based on these assessments, the overall risk level for the introduction of foot-and-mouth disease from Laos into China is determined to be “high”. The effective control of the cross-border spread of foot-and-mouth disease necessitates a comprehensive approach.

## 1. Introduction

### 1.1. An Overview of Foot-and-Mouth Disease

The documentation of Foot-and-Mouth disease (FMD) infection in cattle was recorded in Venice, Italy, in 1514, and it was later confirmed in 1897 that FMD is caused by the Foot-and-Mouth Disease virus (FMDV) [1]. The clinical manifestations and severity of FMD vary depending on the host and viral strain, with common symptoms including blister lesions in the mouth and feet, leading to pain experienced by the affected animals [2]. FMD is a highly contagious transboundary animal disease and falls under the category of endemic diseases [3]. During international trade activities involving animal importation, animals in the incubation period and early stages of the disease pose the greatest risk of infection, significantly impacting animal health and resulting in substantial economic losses, and are considered one of the major animal health burdens worldwide [4,5]. The presence of FMDV within a country can impede the country’s access to international export markets for related animals and their products, affecting the implementation of FMD surveillance and control programs and subsequently resulting in additional economic costs. In developing countries, ruminants and pigs, which play a crucial role in generating income for farmers, are highly susceptible to FMDV. The continuous transmission of FMD will bring them huge economic losses [6]. FMD is endemic in various regions of Asia, Africa, the Middle East, and Latin America [7]. China has classified FMD as a Class I animal disease, while the World Organisation for Animal Health (WOAH) has designated it as a notifiable animal disease. Vaccination, as a key means of preventing and controlling the disease, is still regarded as one of the most effective measures [8,9]. In addition, animal movement control associated with structured FMD control programs, input from robust active and passive surveillance strategies, and the humane culling of infected livestock contributes significantly to the effective management of FMD [10,11,12].

The FMDV is a member of the *Aphthovirus* genus within the *Picornaviridae* family, characterized by its lack of a capsid membrane and symmetrical icosahedral structure in its viral shell [13,14]. There are seven serotypes of the FMDV (O, A, Asia 1, C, SAT 1, SAT 2, and SAT 3) [15]. Currently, in China, types O and A are predominantly prevalent in cases of FMD. Strains Mya-98 and PanAsia of type O, as well as strain Sea-97G2 of type A, are commonly found in countries (regions) neighboring China [13,14,15,16]. The FMDV exhibits remarkable environmental persistence, displaying resistance to various chemicals and desiccation. Its optimal survival conditions are characterized by a pH range of 7–7.5, temperatures below 20 °C, and relative humidity exceeding 55% [17]. The FMDV exhibits a broad host range and is capable of infecting over 70 mammalian species, and it mainly infects cloven-hoofed animals, such as cattle, buffalo, yak, pigs, camels, sheep, and goats [1]. It can also infect wildlife species [18]. The primary modes of transmission for the FMDV are through respiratory and digestive tracts. The movement of infected animals, animals in the incubation period, and carriers of the latent virus play a significant role in both the domestic and cross-border spread of the FMDV [19,20,21]. Healthy animals can contract the FMDV through direct contact with feces and secretions from infected animals, as well as by consuming contaminated water sources or swill. Additionally, the FMDV can also be transmitted through the air, forming an aerosol that can disperse with the wind to places 50 km away, resulting in long-distance and jumping transmission [22]. The jumping transmission refers to FMD outbreaks that suddenly spread over long distances from an infected area to an uninfected area, resulting in the emergence of a new outbreak site.

### 1.2. The Current Situation of FMD in Laos

**Figure 1:**
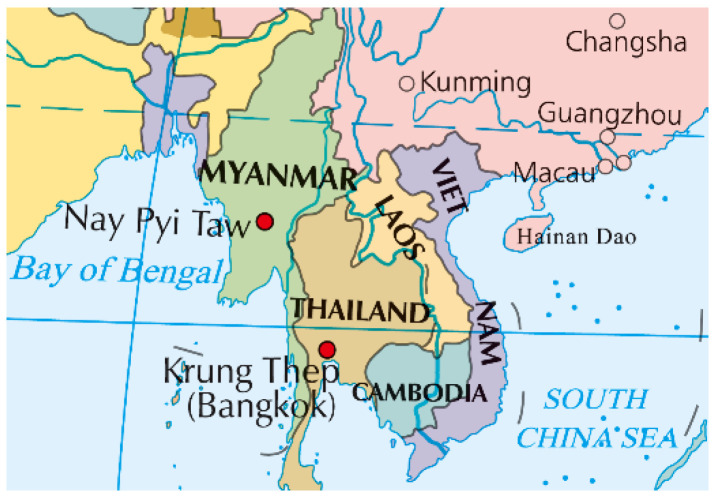
Borders of Laos with neighboring countries (China, Myanmar, Thailand, Cambodia, and Vietnam) (http://bzdt.ch.mnr.gov.cn/browse.html?picId=%224o28b0625501ad13015501ad2bfc0449%22, accessed on 10 May 2024).

Laos, a landlocked country in Southeast Asia, shares borders with Myanmar and Thailand to the west, Cambodia to the south, Vietnam to the east, and China to the north [23] (Figure 1). It serves as a crucial transit route for cross-border animal transport and plays a significant role as an animal cross-border transshipment hub [20,24]. Due to its mountainous terrain, large ruminants are distributed across three main agricultural ecosystems throughout the country. In their study on a massive FMD outbreak in Laos, Perry et al. [25] observed that livestock destined for specific markets followed different trade corridors originating from various source regions. Notably, large ruminants traveled from Cambodia through southern Laos to Thailand. Additionally, piglets and large ruminants from Vietnam had to pass through Laos before reaching Thailand. Even those coming from China needed to traverse northwestern Laos [21]. Eliminating poverty is a top priority for Laos, and measures include increasing income through the raising of water buffalo and cattle [26,27]. The National Agricultural Development Strategy of Laos aims to achieve food security by strengthening agricultural production, with livestock playing an indispensable role [28]. In 2019, the amount of water buffalo meat produced in Laos was four times that of Cambodia, according to FAOSTAT data. As tourism serves as a primary industry in Southeast Asia, the movement of tourists between countries also poses a risk of introducing FMD into these regions [24]. Laos has a relatively limited capacity for animal disease control, lacking the ability to assess animal health and diagnose diseases, exhibiting low efficiency in disease reporting, and facing a lack of knowledge on diseases and biosecurity among farmers and traders, all of which pose further challenges to the control of FMD [29,30]. The biosecurity measures implemented by slaughter facilities in Laos are inadequate, potentially increasing not only the risk of transmitting animal diseases but also that of other zoonotic diseases [31]. Additionally, the vaccination coverage for the FMDV in Laos is limited [32]. Between 2012, 2016 and 2018, the Lao government and international organizations provided funding for the FMDV vaccination in selected provinces. Outside of these provinces, farmers have the option to vaccinate their livestock at their own expense; however, this practice is not widely adopted, and the availability of vaccines remains limited [29,33]. Among the ten countries in Southeast Asia, seven have reported outbreaks of FMD, including Laos. From 1996 to 2001, Laos experienced an outbreak of FMD caused by serotype O FMDV [21]. FMD has been identified as a top priority for control by the WOAH. There is a high seroprevalence of FMD in Laos. A study conducted from March to December 2019 involved the regular collection of a total of 1280 samples of slaughtered animals from six provinces in Laos for the purpose of detecting FMD non-structural protein (NSP) antibodies and determining the natural infection rate. The findings revealed that 22.8% of the samples tested positive for FMD NSP antibodies, with seroprevalence rates among cattle (n = 469), buffalo (n = 214), and pigs (n = 597) being 44.6%, 35.0%, and 1.3%, respectively [19].

### 1.3. The Current Situation of FMD in China

China shares a border with Laos, and from the perspective of spatial distribution characteristics, FMD outbreaks are currently concentrated along the northwest–southeast direction. Specifically, during 1952–1953, the FMDV entered China from the Sino–Myanmar, Sino–India, and Sino–Nepal borders west of the Lancang River in Yunnan, a southwestern border region of China, leading to an FMD outbreak. In 1958, China first reported the prevalence of serotypes O and A of the FMDV in the Xinjiang Uyghur Autonomous Region and serotype Asia 1 in the Yunnan Province. In 1970, the FMDV/O/Cathay topotype was first discovered in Hong Kong, and in 1997, this topotype caused an outbreak in Taiwan. The O/ME-SA/Ind-2001 topotype was first introduced into Laos in 2015, subsequently spreading to Vietnam and Myanmar and entering China in 2017; 27 outbreaks were reported in 2018. Between 2017 and 2021, 19 FMD outbreaks occurred in eight provinces in China. The reasons for this are the uneven development levels of the livestock industry across various regions in China and the continuous outbreaks in neighboring countries (such as India, Southeast Asian countries, and Mongolia) and regions [14,34]. According to statistics published on the official website of the WOAH, more than 500 FMD outbreaks occurred in Southeast Asia between January 2020 and June 2021, posing a significant threat to China’s FMD prevention and control efforts (https://www.woah.org/app/uploads/2024/06/fmd-world-eng-2024.png, accessed on 27 October 2024). The Yunnan Province of China shares a border with Laos, but there is no evidence of FMD outbreaks in the areas adjacent to Laos, as reported by WAHIS (https://wahis.woah.org/#/event-management/, accessed on 27 October 2024). FMD incidents were recorded in Heping, Yuxi, and Qujing within the Yunnan Province in 2013 and 2018; however, these outbreaks occurred far away from the Yunnan–Laos border.

Qualitative risk assessments primarily rely on the subjective judgment and experience of assessors without relying on mathematical models or statistical data. Therefore, they cannot provide a precise quantification of the probability of risk occurrence and the extent of its impact. This can make the assessment results challenging for meeting the requirement for precision in practical applications. However, regarding China’s borders, to date, there is no relevant literature specifically addressing the qualitative risk hazards of FMD transmission from Laos into China. This article aims to provide a reference for this topic.

## 2. Risk Assessment of FMD from Laos to China

The Yunnan Province is situated in the southwestern borderlands of China, sharing borders with Myanmar, Vietnam, and Laos. The interconnectedness of these border areas is facilitated by a network of roads, mountains, and rivers. The local residents collaborate harmoniously in agricultural activities and livestock grazing. The province boasts a total of 28 border crossings and 300 border passages, encompassing 25 border counties and 111 border townships across its eight prefecture-level cities. Due to Yunnan’s exceptional geographical location, it has emerged as a pivotal hub for fostering connectivity between China and Southeast Asian nations. The Yunnan Province shares a 4060 km border, with 710 km of it being shared with Laos. Currently, there are eight designated ports of entry, and the absence of strict border facilities along this borderline poses challenges due to the lack of natural barriers, resulting in occasional instances of illegal trade involving animals and their products [35].

The term “risk” has its origins in the Italian word “RISQUE”. In 1995, the WOAH aimed to address trade frictions and disputes arising from zero-risk decision-making by developing scientifically sound and equitable sanitary measures to regulate trade activities involving animals, plants, and their products [36]. Consequently, the Agreement on the Application of Sanitary and Phytosanitary Measures (referred to as the SPS Agreement) was established, which prominently incorporates risk assessments as a fundamental principle. The SPS Agreement designates the World Organisation for Animal Health (WOAH), Codex Alimentarius Commission (CAC), and Food and Agriculture Organization (FAO) as international standard-setting bodies. The WOAH’s principles of animal health risk analysis, primarily outlined in the Terrestrial Animal Health Code, establish a framework for assessing major animal diseases. These principles not only apply to trade activities involving animals and their products but also extend to decision-making processes related to daily animal health management and the control of significant animal diseases [37]. The WOAH advocates for the use of both qualitative and quantitative methods in risk assessment, emphasizing their flexible selection based on specific circumstances. By implementing an animal health risk analysis system, countries can enhance their early warning capabilities and level of control over animal health and food safety while facilitating international trade in animals and their products [38,39].

The risk assessment in this paper adheres to the animal health risk assessment framework established by the WOAH (Terrestrial Animal Health Code [EB/OL]. (15 March 2024) https://www.woah.org/en/what-we-do/standards/codes-and-manuals/, accessed on 1 May 2024). A qualitative assessment model is employed, which is based on the Covello–Merkhofer model and utilizes descriptors such as “high”, “medium”, and “low”, while a matrix analysis model is also applied [40,41]. Initially, the evaluation of entry and exposure assessments is conducted based on Table 1. Subsequently, these results are integrated with consequence assessments according to Table 2, ultimately leading to a comprehensive assessment conclusion.

### 2.1. Hazards Identification

FMD exists in Laos and is prevalent in most regions, with the vaccines for different viral strains being unable to provide cross-immunity [24]. The absence of stringent vaccination requirements against FMDV in Laos, coupled with the exorbitant costs of vaccines for farmers, has resulted in a significant proportion of farmers refraining from immunizing their livestock. Consequently, this situation poses a substantial risk of animals becoming carriers of FMD. When animals from Laos and their by-products enter China legally, they go through strict quarantine, making it highly unlikely that the FMDV will enter China in this case. The border between China and Laos is long, and there are no natural barriers, so it is not uncommon for animals and their by-products to enter China illegally, during which the FMDV may be introduced into China [42]. The illegal importation of infected animals and their by-products from Laos can easily cause the spread of the FMDV during loading, storage, transportation, and processing, disseminating the virus to various places along the way and posing a significant threat. Infected animals are the most dangerous source of infection and can carry the virus for a long time and excrete it. Animals infected with FMDV may become chronic carriers that do not show clinical symptoms and continue to be infected. Once FMDV is introduced, it may establish a foothold in suitable areas along the border and pose a serious threat to China’s livestock industry through its natural spread and human-mediated transmission.

### 2.2. Entry Assessment

Laos has a relatively limited capacity for animal epidemic prevention and control, leading to frequent outbreaks of FMD within the country. Additionally, there is no mandatory vaccination against the FMDV, resulting in an extremely low vaccination coverage rate that hinders the achievement of herd immunity [24,29]. Consequently, the risk of FMDV transmission during China–Laos trade is comparatively high. China’s requirements for imported animals and their products are strict, and the likelihood of carrying the FMDV is low for legally imported animals and their by-products, with a low risk of FMDV introduction [43].

Frontier trade between border residents refers to the commodity transactions conducted by individuals residing within a 20 km radius of the Chinese land border at officially designated open points or markets, subject to specified limits in terms of the quantity or value traded. Frontier trade between border residents is a distinctive characteristic of China’s border regions, aimed at facilitating commercial activities and enhancing their income levels. Most of this type of trade occurs on a small scale; however, it also involves instances where animals and their by-products from Laos are sold in border markets, thereby increasing the risk of the cross-border transmission of the FMDV. The border between China and Laos is long and difficult to control, and illegal smuggling of animals and their by-products often occurs under the drive of economic interests. The smuggling of animals and their by-products without undergoing quarantine, along with the use of contaminated means of transport, drinking, and feeding tools, as well as bedding and excrement, can serve as potential carriers for the FMDV. Consequently, this significantly escalates the risk of introducing the FMDV into China.

In conclusion, the result of the entry assessment is deemed to be “high”.

### 2.3. Exposure Assessment

The FMDV is highly infectious as it can survive in the environment without animal hosts and exhibit resistance to various environmental conditions, thereby facilitating year-round outbreaks [44]. The FMDV is anticipated to exhibit prolonged viability during the wet season, with survival rates reaching approximately 90% at 16 °C and 86% relative humidity by day 50 [17]. Cows can be infected by semen with or without a diluent, and the infected milk is considered a significant source of infection as dilution and pasteurization do not guarantee the absence of the FMDV [43]. The wool of slaughtered animals carries the virus and may survive at 4 °C for two months [45]. In frozen or fresh-frozen bone-in beef, the survival duration of the FMDV is prolonged. China, as a major country in animal husbandry, boasts a wide range of pig, cattle, and sheep farming and a large number of livestock. There is a vast population of FMD-susceptible animals in the country, among which cattle are highly susceptible [46]. The blistered skin, blistered fluid, blood, urine, feces, milk, exhaled gases, saliva, semen, and viscera of infected animals, as well as the enclosures where infected animals are kept, including contaminated feed, places where infected animals are slaughtered, tools, and discharged sewage may contain live FMDV, and become the source of FMDV infection.

China has established a relatively comprehensive system for the prevention and control of FMD. China adheres to a preventive approach as its primary strategy, implements the regionalized management of FMD, follows the principle of “adapting to local conditions, regional control, and type-specific control”, and actively promotes comprehensive strategies for prevention and control. The State requires units and individuals who raise animals to fulfill their obligations under the Animal Disease Control Law and strictly implement the national FMDV vaccination plan, ensuring that herd immunity density remains above 90% all year round and that the immunization density for livestock and poultry reaches 100%. After vaccination, the regular monitoring of FMDV antibody levels is essential for assessing the immune response. Pigs should undergo antibody testing 28 days post-vaccination, while cattle and sheep should be tested after 21 days. The national requirement is that the number of immune-qualified individuals should account for 70% (or above) of the total immunized population. China possesses comprehensive FMD antibody detection technologies, including liquid-phase block ELISA, solid-phase competitive ELISA, the virus neutralization test (VNT), and the complement binding test (CFT).

In summary, the exposure risk assessment result is deemed to be “medium”.

### 2.4. Consequence Assessment

An outbreak of FMD can lead to a decline in the productivity of animals and even death, as well as increased monitoring and control expenses, culling compensation, trade losses, adverse environmental and social impacts, etc. The morbidity of FMD can reach 100% in susceptible animals, and the mortality rate among adult animals is relatively low (1–5%) but higher in young animals (20% or higher) (https://www.woah.org/en/disease/foot-and-mouth-disease/, accessed on 27 October 2024). FMD significantly impacts livestock production performance. Affected animals experience impaired eating and walking abilities due to the presence of blisters and ulcers in their mouths and hooves, which can have detrimental effects on their growth and reproductive performance. After an outbreak of FMD, the standard procedure to prevent further spread entails the culling of affected animals. However, this approach incurs significant economic losses for both the country and livestock farmers. In response to FMD outbreaks, substantial manpower, material resources, and financial investments are allocated by the government towards vaccination, detection, and virus control efforts. Additionally, disinfection measures for breeding environments and equipment not only result in considerable economic costs but also contribute to environmental pollution.

There is no cross-immunity observed among the different serotypes of the FMDV; thus, the introduction of a new serotype into China will pose greater challenges to the control measures and result in more severe consequences. In response to an outbreak of FMD in a country, other countries typically implement restrictions on the importation of animals and animal products from that specific country, which can have a significant impact on international trade involving animals and animal products. Domestically, an outbreak of FMD would have profound effects on related industries such as meat processing, catering, dairy production, and feed manufacturing sectors while also undermining consumer confidence and purchasing power. Outbreaks of FMD can also have a serious impact on public health, and people can panic about food safety.

In summary, the result of the consequence assessment is deemed to be “high”.

The entry assessment yielded a “High” result, while the exposure assessment resulted in a “Medium” rating. These two values are cross-referenced in Table 1 to determine a unique combined value, ultimately classifying the integrated assessment as “High”. The consequence assessment also resulted in a “High” rating. When evaluated together with the previously combined result of “High” from the entry and exposure assessments, a distinct value is determined through the intersection in Table 2’s matrix. The intersection between “High” and “High” yielded a final rating of “High”. By integrating the results of the entry assessment, exposure assessment, and consequence assessment based on the risk assessment matrix, it was concluded that FMD at the China–Laos border poses a significant risk.

## 3. Methods for Controling FMD from Laos into China

FMD, a highly contagious animal disease of great international concern, has been widely prevalent across the Asian continent [14]. In the absence of specific therapeutic methods, vaccination emerges as the primary choice for prevention, while clinical treatment primarily focuses on symptomatic relief. Laos is a small, impoverished country located on a major pathway for transboundary animal movements within the Greater Mekong Subregion and is closely connected to the border of the Yunnan Province in China [47]. With the advancement of the “Belt and Road” Initiative, frequent trade and personnel exchanges between the two regions have undoubtedly increased the risk of the introduction of animal diseases such as FMD. Therefore, conducting scientific and rational risk assessments is particularly crucial for effectively preventing and controlling the epidemic. Between 2021 and 2024, more than 60,000 outbreaks of FMD occurred in Southeast Asia, with Indonesia being afflicted by the most severe outbreaks (https://wahis.woah.org/#/dashboards/qd-dashboard, accessed on 27 October 2024). This presented a significant challenge to China’s efforts to control FMD. In the course of the analysis, we opted for a qualitative assessment approach, owing to the fact that qualitative methods are more intuitive, yield results that are readily comprehensible and can, therefore, more directly inform the formulation of import quarantine policies.

Risk assessment plays a pivotal role in the prevention and control of animal diseases. Several researchers have conducted studies on the risk analysis of foot-and-mouth disease using diverse methodologies. Zheng Teng et al. [48] conducted a qualitative assessment of seven potentially hazardous animal diseases, emphasizing the high and unacceptable risk of FMD introduction, suggesting that beef and its products could be imported conditionally. Moreover, Yang Honglin et al. developed a stochastic model to assess the risk of FMD introduction through live cattle smuggling across the China–Myanmar border in Yunnan, using a scene tree analysis to quantitatively evaluate the probability of introducing FMD via one head of smuggled cattle (0.81%) [49]. These findings provide significant data support for efforts aimed at preventing and controlling FMD. China is adjacent to the northern border of Laos. Emily Gee collected 2663 serum samples of cattle and buffalo from provincial abattoirs in northern Lao between November 2021 and December 2022 and invested serological data of the FMDV [47]. Samples were tested for specific antibodies directed against the FMD non-structural protein (NSP) to determine the proportion of animals exposed to the FMD virus, and the positive rate reached up to 46% overall. So, FMD surveillance and risk assessment are essential for disease control.

Over the years, significant measures have been taken by the Chinese government to control FMD transmission, such as implementing compulsory immunization plans and emergency disposal strategies for epidemics. Mandatory vaccination against the FMD O serotype is required for all pigs, while cattle and sheep must be vaccinated against FMD serotype O with Asia 1 vaccines. Serological tests, virus neutralization tests, and enzyme-linked immune sorbent assay (ELISA) methods are utilized to evaluate the immune efficacy of FMD vaccines. Between 2016 and 2019, Yang Jingzhu et al. [50] conducted a detailed study on cattle and sheep epidemics in three counties of Lincang City, finding that imported cattle had low immunization levels and high rates of silent virus carriage. As of 2021, an immunization survey of FMD in border areas of the Yunnan Province showed that the immunization level of the FMDV in border areas was still inadequate, with significantly higher antibody-positive rates in imported cattle compared to local cattle, indicating a risk of virus introduction [51]. Therefore, enhanced serological monitoring and vaccination are required.

The widespread prevalence of FMD in China and neighboring Laos poses severe challenges to the development of livestock farming in both countries. In China, outbreaks of FMD have not only resulted in significant livestock losses and huge economic damage but have also increased the difficulty of epidemic prevention work and the demand for vaccines. Furthermore, due to virus mutations and the introduction of foreign strains, prevention and control efforts have become even more complex and challenging. Laos, as a high-incidence area for FMD, faces the risk of its outbreaks spreading to China through trade, cross-border wildlife activities, and smuggling, further intensifying China’s prevention and control pressures. This not only impacts China’s livestock farming industry but also poses a serious threat to China’s food safety and public health, affecting people’s quality of life and health levels. These include inadequate prevention and control capabilities, the lengthy and unshielded border, frequent small-scale animal trade across the border, illegal smuggling activities, the risk of transmission from wildlife, and insufficient quarantine and inspection measures. Drawing upon previous advancements in risk assessment, this study conducted a qualitative risk assessment of the likelihood of FMD transmission from Laos to China, utilizing three dimensions: an entry assessment, exposure assessment, and consequence assessment. The assessment concluded that the risk is classified as “high”. This research aims to provide a qualitative reference for relevant prevention and control efforts, thereby facilitating China’s effective response to the risk of animal disease introduction, including FMD. Based on this risk assessment, we recognize that more comprehensive and in-depth measures are necessary to effectively prevent and control FMD. As tourism serves as a primary industry in Southeast Asia, the movement of tourists between countries also poses a risk of introducing FMD into these regions.

## 4. Suggestions for Preventing FMD Transmission from Laos into China

As a result of these findings, Laos and China need to work together to improve FMD surveillance and control to reduce the risk. To achieve this goal, we should further strengthen the cooperation between China and Laos in the prevention and control of FMD. This includes formulating epidemic prevention and control strategies that are suitable for the national conditions of both countries; encouraging and supporting Chinese enterprises and personnel to assist in FMD prevention and control efforts in Laos; and jointly establishing standardized large-scale farms, quarantine stations, and vaccine research and development units. Regularly conducting veterinary technology training courses in border areas can enhance the skills of veterinary technicians, livestock farmers, and government personnel involved in the livestock industry from both China and Laos. FMD prevention and control capabilities can also be enhanced through technical exchanges and sharing experiences. Robust data information sharing systems can be established between both governments, conducting regular liaison meetings for exchanging information on animal epidemics and prevention measures, thereby jointly safeguarding public health security. Actively establishing cross-border animal epidemic monitoring stations while launching projects for overseas epidemic prevention and control can also strengthen FMD management.

On this basis, we should strengthen the capacity for FMD monitoring, including early warnings and responses in the China–Laos border areas. Regular monitoring of susceptible animals carrying the FMDV in the border areas between China and Laos is conducted every year to keep up to date with the FMDV epidemic situation in the border areas. Emphasis is placed on enhancing the monitoring of border ports, processing and holding facilities of susceptible animals and their by-products, and the surrounding areas. Public awareness should also be enhanced by strengthening publicity efforts regarding FMD control work and promoting knowledge about FMD control measures. The widespread vaccination of susceptible animals in threatened areas along the Chinese border, the continuous enhancement of the epidemic prevention and control system and technologies, and providing “vaccination fee subsidies after vaccination” for large-scale livestock farmers are all effective measures to mitigate disease transmission. Strengthening the construction of an epidemic early warning system in border areas while formulating emergency response plans tailored to local conditions for the effective management of FMD outbreaks is also essential. Enhancing responsiveness capabilities during sudden events can ensure prompt and effective actions when epidemics occur.

Furthermore, we should further strengthen the quarantine inspection at entry ports to strictly prevent the illegal entry of animals and their products; ensure adequate funding for personnel, testing, and monitoring activities; enhance the capacity of entry port inspection and quarantine by upgrading testing equipment both at entry ports and laboratories; improve the expertise of personnel at entry ports in conducting inspections; and enhance the detection technology used and proficiency level of laboratory staff. The inspection and quarantine supervision at ports should be strengthened, with targeted inspections conducted on suspicious animals and products. Efforts to combat the illicit importation of animals and their products while simultaneously enhancing border inspections and patrols to prevent the unauthorized entry of animals carrying FMDV should also be strengthened.

Lastly, for the protection and management of wildlife, the illegal hunting and trade of wildlife, particularly those potentially carrying the FMDV, should be prohibited, rigorously combating the illicit trafficking of wildlife from neighboring countries such as Laos. Enhancing the safeguarding and administration of wildlife habitats can minimize opportunities for livestock–wildlife interactions. Proactively monitoring wildlife for the FMDV presence, and if any infected animals are detected, restricting their movement range, establishing quarantine zones, and conducting weekly surveillance in the affected area while extending monitoring activities by a few kilometers to prevent disease transmission are essential steps.

In summary, the effective control of the cross-border spread of FMD necessitates a comprehensive approach to prevention and control measures. Only through the comprehensive strengthening of preventive and control efforts can the dissemination and outbreak of FMD be effectively managed.

## 5. Conclusions: Moving Forward

Laos is relatively weak in animal disease prevention and control, and FMD occurs frequently. Given the extensive border between China and Laos without natural barriers, effectively controlling the cross-border transmission of animal diseases in these areas is challenging. The illegal entry of animals and their products, as well as the unrestricted movement of wild and domestic animals across borders, greatly facilitates the spread of animal diseases across borders. After conducting an entry assessment, exposure assessment, and consequence assessment, it was determined that there is a “high” risk of FMD spreading from Laos to China. In order to prevent the spread of FMD from the China–Laos border area into China and ensure the safety of domestic agriculture and meat production, it is recommended that cooperation between China and Laos is enhanced to prevent and control the cross-border transmission of FMD. This can be achieved by strengthening monitoring, early warning, and response capabilities for FMD in the China–Laos border area, as well as implementing comprehensive control measures such as enhancing animal-entry quarantine procedures and restricting the cross-border movement of animals. To enhance the precision of assessing the risk of FMD transmission from Laos to China, it is imperative to establish a quantitative risk assessment model in the future. This is because this model will comprehensively consider diverse factors, encompassing animal and product movement, the ecological environment in border areas, the prevalence of FMD in Laos, and China’s prevention and control capabilities. By conducting a scientific and systematic evaluation of cross-border transmission risk for FMD, risk assessments could provide more accurate predictions regarding transmission trends and serve as a scientific foundation for formulating more effective strategies for prevention and control.

## Figures and Tables

**Table 1:** Comprehensive evaluation matrix of entry and exposure assessments.

		Exposure Assessment					
		Negligible	Very Low	Low	Medium	High	Very High
Entry assessment	very high	medium	medium	high	high	very high	very high
	high	medium	medium	medium	high	high	very high
	medium	low	low	medium	medium	high	high
	low	low	low	low	medium	medium	high
	very low	very low	very low	low	low	medium	medium
	negligible	negligible	very low	low	low	medium	medium

**Table 2:** Comprehensive evaluation matrix of entry, exposure, and consequence assessments.

		Consequence Assessment					
		Negligible	Very low	Low	Medium	High	Very High
Entry and exposure assessment	very high	negligible	low	medium	medium	high	very high
	high	negligible	very low	medium	medium	high	high
	medium	negligible	very low	low	medium	medium	medium
	low	negligible	negligible	very low	low	low	low
	Very low	negligible	negligible	negligible	very low	very low	very low
	negligible	negligible	negligible	negligible	negligible	negligible	negligible

## Data Availability

No new data were created or analyzed in this study. Data sharing is not applicable to this article.
